# Using audiovisual intervention to reduce anxiety and improve image quality in pediatric magnetic resonance imaging

**DOI:** 10.1007/s00247-025-06276-5

**Published:** 2025-06-05

**Authors:** Seçil Oktay, Türker Söğütlüler, Sibelnur Avcil, Ceren Akçanal Şeker, Hatice Öner, Hakan Öztürk, Ayşe Tosun, Yelda Özsunar

**Affiliations:** 1 Aydın Gynecology and Pediatrics Hospital, Aydin, Turkey; 2 Adnan Menderes University, Aydin, Turkey

**Keywords:** Anxiety, Auditory perception, Child, Magnetic resonance imaging, Image enhancement, Persuasive communication, Visual perception

## Abstract

**Background:**

Magnetic resonance imaging (MRI) is a crucial diagnostic modality for pediatric patients because it avoids ionizing radiation. However, the confined space and loud noises inherent to MRI can trigger anxiety in children, potentially compromising their cooperation and image quality.

**Objective:**

To evaluate the effectiveness of an audiovisual preparatory intervention in reducing anxiety and improving MRI image quality in children.

**Materials and methods:**

In this randomized controlled trial, 48 children aged 7–11 years referred for MRI were randomly assigned to either an experimental group (*n*=24) or a control group (*n*=24). The experimental group viewed a child-friendly preparatory film prior to the MRI. Anxiety was assessed using the State-Trait Anxiety Inventory for Children before and after MRI. Image quality was evaluated independently by blinded radiologists using standardized scoring criteria.

**Results:**

There were no significant differences in baseline state anxiety (*P*=0.790) or trait anxiety (*P*=0.414) between groups. Post-MRI state anxiety scores were significantly lower in the experimental group (31.17±8.78) compared to the control group (37.90±6.51; *P*=0.004). Post-MRI trait anxiety scores remained statistically similar (*P*=0.491). Image quality scores were significantly higher in the experimental group than in the control group (*P*=0.005).

**Conclusion:**

Audiovisual preparatory interventions significantly reduce state anxiety and enhance image quality in pediatric MRI without affecting trait anxiety. These findings support the integration of non-pharmacological, child-centered preparatory tools into routine MRI protocols to improve patient cooperation and diagnostic outcomes.

**Graphical Abstract:**

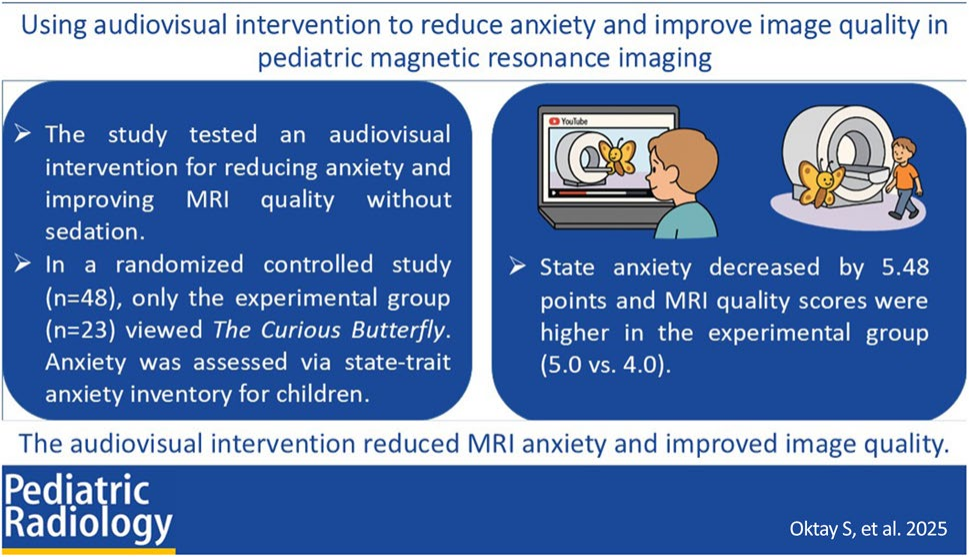

**Supplementary Information:**

The online version contains supplementary material available at 10.1007/s00247-025-06276-5.

## Introduction

Magnetic resonance imaging (MRI) is an essential diagnostic tool frequently utilized in pediatric populations due to its lack of ionizing radiation and its ability to provide high-resolution anatomical details, surpassing many other imaging modalities [1, 2]. Despite these advantages, the nature of the procedure presents significant challenges for pediatric patients. The long, narrow, and enclosed structure of the MRI scanner, coupled with its loud and repetitive noises, often triggers anxiety, fear, and agitation, particularly in children and claustrophobic individuals. Additionally, the lack of familiarity with the medical personnel, the intimidating environment, and insufficient preparatory information exacerbate these issues, reducing cooperation and adversely affecting image quality [1, 3].

Motion artifacts caused by a child’s inability to remain still during the scan can compromise the diagnostic value of MRI images, making immobilization critical for successful imaging [2, 4]. To address this challenge, sedation or general anesthesia is frequently employed, particularly in younger children under the age of seven, to ensure immobility and improve imaging outcomes [5]. However, these pharmacological interventions come with notable risks and logistical challenges. Sedation and anesthesia carry potential complications such as respiratory depression, cardiac rhythm disturbances, gastrointestinal side effects, and prolonged recovery times, including psychomotor agitation or excessive drowsiness [6, 7]. Moreover, these interventions increase healthcare costs due to the need for specialized personnel, equipment, and extended hospital stays [8, 9].

Although chloral hydrate, midazolam, and other agents are commonly used for sedation in pediatric imaging, they are not without limitations. Chloral hydrate, for instance, is associated with respiratory distress, insufficient sedation, and delayed recovery, while benzodiazepines and other sedatives often fail to achieve adequate immobilization during MRI [7, 10]. Furthermore, sedation procedures require advanced monitoring systems and resuscitation equipment compatible with the MRI environment, adding to the complexity and cost [11]. Given these limitations, there is an urgent need to explore alternative, non-pharmacological methods that can effectively reduce anxiety and ensure patient compliance without the inherent risks and financial burden of sedation or anesthesia [12].

The present study aims to evaluate the efficacy of audiovisual artistic interventions, such as videos and fairy tale therapy, in reducing pre-MRI anxiety and enhancing the quality of radiological images. By employing these alternative strategies, this study seeks to minimize reliance on sedation, reduce the associated risks and costs, and improve the overall MRI experience for pediatric patients. Ultimately, these approaches could pave the way for safer and more patient-centered imaging practices in pediatric radiology.

## Materials and methods

### Study design and setting

This prospective, randomized controlled trial was conducted between January and September 2023 at Aydın Adnan Menderes University Faculty of Medicine. The necessary ethics committee approval was obtained for the study (decision number, E-50107718–050.99–3512). The assessments and evaluations were conducted by a multidisciplinary team consisting of S.O., a pediatric neurologist with 5 years of experience; T.S., a communication scientist with 8 years of experience; S.A., a child psychiatrist with 14 years of experience; C.A.Ş., a child psychiatrist with 3 years of experience; H.Ö., a psychiatric nurse with 11 years of experience; H.Ö., a biostatistician with 11 years of experience; A.T., a pediatric neurologist with 20 years of experience; and Y.Ö., a neuroradiologist with 25 years of experience.

The study included outpatients who were referred directly to the pediatric neurology clinic or referred from the general pediatric clinic, who had normal mental and consciousness examinations, no acute neurological pathology detected during physical examination, and who had not previously undergone an MRI scan. Patients with elective cranial MRI indications, such as investigation of the etiology of previous seizures or chronic headaches, were referred to the department of child and adolescent mental health diseases prior to imaging. The sociodemographic data form created by the researchers, by the child psychiatrist and the clinician scale, were applied to determine that the children did not have any psychopathology. The participants included in the study were divided into a case group and a control group by simple randomization. The case group was shown the “Curious Butterfly” video by the child psychiatrist. The families were told that the children would watch the video an average of five times until the MRI was taken. All patients underwent routine non-contrast cranial MRI examinations. Imaging was performed on a 3.0-T scanner (GE SIGNA™ Pioneer, GE Healthcare, Chicago, IL) using a standard head coil in the university’s radiology department. The imaging protocol included the following conventional sequences: axial T1-weighted spin echo, axial and coronal T2-weighted turbo spin echo, axial fluid-attenuated inversion recovery (FLAIR), axial susceptibility-weighted imaging (SWI), and axial diffusion-weighted imaging (DWI) with apparent diffusion coefficient (ADC) map reconstruction. All sequences were acquired with a slice thickness of 5 mm, and imaging parameters—repetition time (TR), echo time (TE), matrix, and field of view (FOV)—were optimized according to routine clinical practice. The FLAIR sequence was acquired in the axial plane, with a TR of 8,500 ms, TE of 117 ms, slice thickness of 5 mm, matrix size of 184 × 256, and a FOV of 240 × 240 mm. An inversion recovery (IR) time of 2,500 ms was also applied for this sequence. The T2 turbo spin echo (TSE) sequence was obtained in both axial and coronal planes, with parameters including TR of 2,500 ms, TE of 100 ms, 5 mm slice thickness, a matrix of 360 × 288, and FOV of 220 × 220 mm. The T2*/SWI sequence was performed in the axial plane, with TR of 360 ms, TE of 10 ms, and 5 mm slice thickness. DWI was also acquired axially, using TR of 5,202 ms, TE of 78 ms, slice thickness of 5 mm, and a FOV of 230 × 240 mm; ADC maps were included in the evaluation. The T1 spin echo (SE) sequence was obtained in both axial and sagittal planes, with TR of 2,325 ms, TE of 24 ms, slice thickness of 5 mm, matrix size of 240 × 240, and FOV of 300 × 224 mm. This standardized protocol applied uniformly. The total scan time was approximately 20–25 min per patient, and none of the patients received sedation. All sequences were reviewed to assess image quality. State-Trait Anxiety Inventory scales were filled out by the children in the case and control groups on the same day before and after imaging.

### Sociodemographic data form

Age, sex, education status, number of siblings, additional physical disorders, and medication use were questioned in the sociodemographic data form created by the researchers.

### Sample size and participants

The required sample size was calculated based on the study by Tazegül et al. [13], with an effect size of *d* = 1.2477, a type I error rate of *α* = 0.05, and a power level (1*-β*) of 0.95, resulting in a minimum of 36 participants (18 per group). To account for potential dropouts, 50 children were initially recruited and randomly assigned to the experimental (*n* = 25) and control (*n* = 25) groups. Two participants from the experimental group were excluded for not completing the final assessment, resulting in a final sample of 48 children (23 experimental, 25 control). Two participants were unable to complete their full participation due to family-related time constraints; however, this occurred independently of the study procedures.

### Inclusion and exclusion criteria

Participants were included in the study if they were between 7 and 11 years old, had no prior exposure to MRI, had no psychiatric diagnosis or clinical mental retardation as determined by diagnostic interviews, and provided voluntary participation with consent from their legal guardians. None of the children included in the study required sedation during the MRI; therefore, sedation was not administered. Exclusion criteria encompassed intense anxiety requiring sedation for other medical reasons, hospitalization in general or intensive care units, referral from the emergency department, or the presence of trauma, moderate-to-severe illness, shock, or coma.

### Psychiatric assessment and anxiety evaluation

The Schedule for Affective Disorders and Schizophrenia for School-Age Children-Present and Lifetime Version (K-SADS-PL) is a diagnostic semi-structured interview tool which is used to determine past and current psychiatric psychopathologic disorders in children and adolescents based on DSM-III and DSM-IV criteria [14]. In 2016, it was revised according to DSM-5 criteria by Kaufman et al. [14]. The validity and reliability study for the xxxxxxx adaptation of the version updated according to DSM-5 diagnoses was conducted by Ünal et al. [15]. K-SADS-PL-DSM-5-T is applied by interviewing the parents and the child, and at the end, scoring is made based on the information received. The schedule can detect mood disorders, anxiety disorders, disruptive behavior disorders, psychotic disorders, tic disorders, elimination disorders, substance abuse, and eating disorders [15].

### Turkish version of the State and Trait Anxiety Inventory for children

The State-Trait Anxiety Inventory was developed by Spielberger et al. in 1970 and adapted to xxxxxxx society by Öner and Le Compte in 1983. It is a self-reported Likert-type scale that measures state and trait anxiety levels separately. State-Trait Anxiety Inventory, consisting of 20 questions, is a very sensitive tool in evaluating sudden changes in emotional reactions. The Trait Anxiety Scale, which also consists of 20 questions and is in the second part of the inventory, aims to measure the continuity of anxiety that the person generally tends to experience. It is a 4-point scale ranging from “not at all” to “completely.” The scale contains direct and inverted questions. Direct questions express negative emotions, while inverted questions express positive emotions. The total score obtained from both scales varies between 20–80. High scores indicate high anxiety level; low scores indicate low anxiety level [16].

### Intervention

The experimental group was shown a custom-designed audiovisual media product prior to the MRI examination. This short film, titled *The Curious Butterfly*, was specifically developed for the study to help pediatric patients perceive the MRI process as less intimidating. The storyline follows a child and a curious butterfly as they journey through hospital corridors to the MRI scanner, which is portrayed as a large photo camera.

**Fig. 1:**
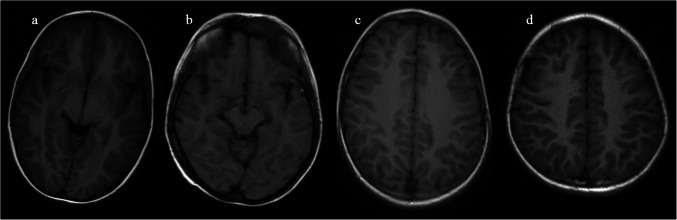
Axial unenhanced T1-weighted magnetic resonance images from control (**a**, **b**) and intervention (**c**, **d**) patients rated as 2 in an 8-year-old girl (**a**), 3 in an 8-year-old boy (**b**), 4 in a 7-year-old girl (**c**), and 5 in an 8-year-old boy (**d**) on a 5-point scale, where higher scores reflect better image quality

The video features vibrant animations, child-friendly narration, soft background music, and a calming atmosphere, aiming to transform the MRI procedure into an engaging and imaginative experience. As the child lies still inside the scanner, the butterfly stays nearby, offering reassurance through visual animation and ambient sound. The audiovisual content was produced with support from the university’s Faculty of Communication, while voiceover production was carried out in collaboration with the hospital’s radiology department. The video was delivered via YouTube [17], and a representative screenshot is shown in Fig. 1. During the MRI scan, the butterfly imagery was projected directly onto the MRI device using Troykamed technology (https://troykamed.com/), integrating the audiovisual experience into the procedure. In contrast, the control group underwent standard MRI without exposure to the audiovisual intervention. Following the scan, all participants completed the State-Trait Anxiety Inventory once again to assess post-intervention anxiety levels.

### Magnetic resonance image quality evaluation

MRI images were evaluated in a blinded manner by two independent reviewers. One reviewer was a radiology resident in training, and all assessments were subsequently reviewed and confirmed by a senior faculty member. One board-certified neuroradiologist with more than 20 years of experience in pediatric brain imaging and a pediatric neurologist independently evaluated image quality. Radiologists used a 5-point scale, where a score of 5 indicated “very good” image quality with minimal motion artifacts, and a score of 1 indicated “very poor” quality with significant artifacts. Image quality was evaluated for each MRI sequence described in the “Materials and methods” section. The assessment focused on the presence of motion artifacts, the clarity of anatomical details, the degree of gray–white matter differentiation, and the overall diagnostic adequacy of the images. Agreement between the two reviewers was analyzed using weighted kappa statistics. A nearly perfect agreement was observed (*κ* = 0.91; *P* < 0.001). Average image quality scores for each group were calculated and compared.

### Statistical analysis

Data were analyzed using IBM SPSS Statistics for Windows, version 26.0 (IBM Corp., Armonk, NY). The Kolmogorov–Smirnov test assessed the normality of quantitative variables. Descriptive statistics were presented as mean ± standard deviation for normally distributed variables, median (25th–75th percentile) for non-normally distributed variables, and frequency (%) for categorical variables. Independent group comparisons were conducted using the independent samples *t*-test for normally distributed variables and the Mann–Whitney *U* test for non-normally distributed variables. Categorical variables were analyzed using chi-square independence tests. A *P*-value of < 0.05 was considered statistically significant.

## Results

### Demographic characteristics

The demographic characteristics of the experimental and control groups are summarized in Table 1. The mean age of participants in the experimental group was 8.69 ± 1.26 years, compared to 8.96 ± 1.51 years in the control group. Statistical analysis revealed no significant difference in age between the two groups (*P* = 0.516). Sex distribution was also homogeneous across groups, with no significant differences observed (*P* = 0.413).

**Table 1:** Demographic characteristics of the experimental and control groups

Groups	Experimental	Control	*P*-value
Age (years)	8.69 ± 1.26	8.96 ± 1.51	0.516^a^
Sex			
Female	10 (43.5)	7 (28.0)	0.413^b^
Male	13 (56.5)	18 (72.0)	

^a^Independent samples *t*-test; ^b^chi-square independence analysis, descriptive statistics are expressed as mean ± standard deviation or frequency (%)

### Anxiety scores

The initial and final state and trait anxiety scores are presented in Table 2. There were no significant differences between the experimental and control groups in terms of initial state anxiety (*P* = 0.790) and trait anxiety (*P* = 0.414). However, the final State-Trait Anxiety Inventory scores were significantly lower in the experimental group compared to the control group (31.17 ± 8.78 vs. 37.90 ± 6.51, *P* = 0.004). Conversely, no statistically significant difference was observed between the groups for final trait anxiety scores (*P* = 0.491).

**Table 2:** Comparison of State-Trait Anxiety Inventory scores

Groups	Experimental	Control	*P*-value
State anxiety (initial)	36.65 ± 8.98	37.28 ± 7.34	0.790
State anxiety (final)	31.17 ± 8.78	37.90 ± 6.51	**0.004**
Trait anxiety (initial)	38.72 ± 8.29	40.71 ± 8.49	0.414
Trait anxiety (final)	38.91 ± 6.61	40.39 ± 7.98	0.491

Independent samples *t*-test, descriptive statistics are expressed as mean ± standard deviation

Values in bold indicate statistically significant differences (*P* 0.05)

### Magnetic resonance image quality

The scores of the observers’ assessment of MRI quality in the experimental and control groups are given in Table 3.

**Table 3:** Observers’ assessment scores for magnetic resonance image quality

Experimental	Observer 1	Observer 2	Control	Observer 1	Observer 2
1	5	5	1	4	4
2	5	5	2	4	4
3	2	4	3	2	4
4	5	5	4	4	4
5	2	4	5	5	5
6	5	5	6	4	4
7	5	5	7	4	4
8	4	4	8	4	4
9	4	4	9	4	4
10	5	5	10	3	3
11	4	4	11	3	3
12	3	3	12	4	4
13	4	4	13	3	3
14	5	5	14	4	4
15	5	5	15	5	5
16	5	5	16	4	4
17	5	5	17	2	2
18	3	3	18	5	5
19	5	5	19	2	2
20	5	5	20	3	3
21	5	5	21	5	5
22	5	5	22	4	4
23	4	4	23	4	4
			24	3	3
			25	2	2

The comparison of MRI quality scores between the experimental and control groups is presented in Table 4. The experimental group demonstrated significantly higher MRI quality scores compared to the control group (median, 5.0 (4.0–5.0) vs. 4.0 (3.0–4.0); *P* = 0.005)

**Table 4:** Comparison of magnetic resonance image quality

Groups	Experimental	Control	*P*-value
MRI quality score	5.0 (4.0–5.0)	4.0 (3.0–4.0)	**0.005**^a^

^a^Mann-Whitney *U* test, descriptive statistics are expressed as median (25th-75th percentile), *MRI* magnetic resonance imaging

Values in bold indicate statistically significant differences (*P* 0.05)

## Discussion

This study provides a significant contribution to the existing literature by exploring innovative, non-pharmacological strategies to address the challenges of pediatric MRI procedures. While sedation and general anesthesia remain the conventional approaches for ensuring immobility and improving image quality, these methods are associated with considerable risks, side effects, and increased healthcare costs. By focusing on audiovisual artistic interventions, this research presents alternative approaches that have the potential to mitigate pre-procedural anxiety and enhance diagnostic outcomes without relying on pharmacological solutions.

The study bridges a critical gap in pediatric radiology by integrating therapeutic storytelling, bibliotherapy, and guided imagery into MRI preparation, emphasizing their effectiveness in reducing anxiety and fostering cooperation in children. Furthermore, the findings contribute to the growing body of evidence on the use of creative and engaging methods, such as adventure-based narratives and visual-auditory stimuli, to transform intimidating medical environments into child-friendly experiences. These approaches not only alleviate the psychological burden of MRI for pediatric patients but also address broader healthcare priorities by reducing the dependency on sedation, minimizing associated risks, and lowering procedural costs.

This research establishes a practical and scalable framework for incorporating artistic interventions into routine pediatric imaging practices. By reframing the MRI experience as an engaging and less stressful process, the study introduces a paradigm shift in how anxiety and compliance are managed during diagnostic procedures. These findings have the potential to inspire further research into child-centered, non-invasive methodologies, ultimately enhancing patient care and optimizing outcomes in pediatric radiology.

Several non-invasive strategies have been proposed to address pre-MRI anxiety and enhance patient cooperation. Preparatory interventions such as educational videos explaining the MRI process, familiarization with the MRI environment and equipment, breathing exercises, storytelling, and guided imagery have shown promise in alleviating fear and anxiety in children [18–20]. Additionally, the use of audiovisual systems during MRI procedures—such as allowing children to watch videos or listen to music—has been found to reduce anxiety and improve the overall patient experience [21]. Art therapy, particularly bibliotherapy and fairy tale-based storytelling, is another effective approach for engaging children in a therapeutic and creative process. By shifting the child’s focus from the intimidating aspects of the MRI to imaginative narratives, these interventions foster a calming and engaging environment [22, 23].

Research suggests that adventure-based narratives, especially those involving relatable child protagonists, resonate deeply with pediatric patients, helping to alleviate stress and fear [24, 25]. Integrating such narratives into audiovisual interventions can transform the MRI experience from a stressful medical procedure into an enjoyable and immersive activity.

Ensuring immobility during MRI is critical for obtaining high-quality imaging in pediatric patients. While sedation and general anesthesia are frequently employed to achieve this, these methods are associated with significant risks, side effects, and financial costs. This study supports the growing interest in alternative, non-pharmacological strategies to reduce pre-MRI anxiety and enhance patient cooperation, thereby minimizing the reliance on sedation.

A variety of approaches, such as pre-scan demonstrations, audiovisual aids, explanatory videos, guided imagery, and storytelling, have been shown to effectively reduce anxiety and improve patient compliance. For instance, Carter et al. [21] found that pre-scan demonstrations for children aged 3–8 years significantly reduced sedation requirements, while audiovisual systems embedded in MRI devices have demonstrated a 34% reduction in sedation needs among certain age groups [22, 23]. Similarly, guided imagery and music interventions have exhibited moderate success in decreasing anxiety and improving cooperation [24]. These findings align with our results, where the experimental group exposed to audiovisual media demonstrated significantly lower anxiety levels and improved MRI quality compared to the control group (*P* = 0.004; *P* = 0.005).

Bibliotherapy and storytelling also play a pivotal role in creating an anxiety-free experience for children during MRI. Techniques like fairy tale therapy help redirect the child’s focus from the intimidating aspects of the procedure to engaging narratives, significantly reducing anxiety [26, 27]. Studies such as Sarıalioğlu et al. [28] and Amer et al. [29] confirm the effectiveness of storytelling in alleviating preoperative fear and anxiety, further supporting our findings.

Effective communication with both patients and families is another critical element in reducing anxiety. Nakada et al. [30] highlighted the importance of patient-centered communication techniques to mitigate stress caused by MRI’s confined and noisy environment. In line with these findings, our study emphasizes the necessity of training healthcare personnel to adopt patient-oriented strategies to enhance the MRI experience for children.

The integration of technology-based interventions, such as virtual reality and audiovisual systems, offers promising opportunities for transforming pediatric healthcare practices. Studies have demonstrated that virtual reality glasses showing animated educational content significantly diminish preoperative anxiety in children [31]. Similarly, audiovisual systems can reduce the need for sedation by up to 18% and decrease scan duration [22, 23, 32].

Our findings suggest that audiovisual interventions can promote a more supportive and engaging hospital environment by not only alleviating anxiety but also improving image quality. This aligns with existing research advocating for the use of complementary techniques, such as hypnosis and guided imagery, to redirect attention and improve patient cooperation during medical procedures [33–36]. These methods reduce the emotional burden of MRI while minimizing sedation-related risks, as highlighted in previous studies [7, 37–39].

## Conclusion

This study underscores the effectiveness of alternative methods, particularly audiovisual storytelling, in reducing anxiety and improving MRI image quality in pediatric patients. By minimizing the need for sedation, these approaches address both the emotional and physical needs of young patients, while fostering a more positive and child-friendly medical environment.

The results contribute to the growing body of literature emphasizing non-pharmacological interventions in pediatric care and highlight the transformative potential of innovative practices in radiology. By integrating creative approaches like storytelling and guided imagery, healthcare providers can significantly improve patient experiences, outcomes, and overall service quality.

Future research should continue exploring these methods to refine their application and further revolutionize pediatric healthcare practices. The integration of art therapy and technology-based solutions into routine care has the potential to set new standards for managing anxiety in children undergoing medical procedures, ultimately enhancing both patient satisfaction and clinical outcomes.

## Supplementary Information

Below is the link to the electronic supplementary material.

Supplementary file1 (MP4 254272 KB)

## Data Availability

The dataset is available at"https://figshare.com/articles/dataset/merakl_kelebek_sav/28882808?file=54,017,522.
